# Rare Earth Doped Silica Nanoparticles *via* Thermolysis of a Single Source Metallasilsesquioxane Precursor

**DOI:** 10.1038/srep45862

**Published:** 2017-04-05

**Authors:** Gemma-Louise Davies, John O’Brien, Yurii K. Gun’ko

**Affiliations:** 1 Department of Chemistry, University of Warwick, Coventry CV4 7AL, UK; 2 School of Chemistry and CRANN Institute, Trinity College Dublin, Dublin 2, Ireland; 3 St. Petersburg National Research University of Information Technologies, Mechanics and Optics, 197101, St. Petersburg, Russia

## Abstract

Rare earth metal doped silica nanoparticles have significant advantages over traditional organic dyes and quantum dots. Silsesquioxanes are promising precursors in the production of silica nanoparticles by thermolysis, due to their structural similarities with silica materials. This manuscript describes the production of a new Eu^3+^-based metallasilsesquioxane species and its use as a single source precursor in the thermolytic production of luminescent rare earth metal doped silica nanoparticles with characteristic emission in the visible region of the spectrum.

Luminescent nanoparticles hold great promise as biomarker tools in the emerging field of nanomedicine. Whilst organic dyes and quantum dot based materials possess useful properties, their downsides, such as the ease of photobleaching of organic dyes or the toxicity of heavy metal containing quantum dots, can be severely limiting to their practical application. Rare earth (RE) metal doped oxide nanomaterials on the other hand, may offer advantages over these materials, due to their long luminescence lifetimes, resistance to photobleaching and sharp emission profiles. The incorporation of RE ions into silica and silicates is of interest for a wide variety of applications, including optics, catalysis and biomedicine^1^,^2^,^3^.

Silica-based RE-doped particles have great potential as luminescent probes for biological systems, due to their small sizes, (potentially) strong emission properties and silanol surfaces, which allow further modification with ease. The most common route to such materials is the grafting or incorporation of a lanthanide complex, whose emission properties are enhanced through an “antenna” effect^4^,^5^,^6^. Rare earth ions and oxides can alternatively be incorporated directly into silica nanoparticles or bulk silicate structures, including zeolites, using solution chemistry processing or non-solution approaches. Solution chemistry techniques generally involve the addition of rare earth salts to acid or base catalyzed sol-gel reactions, yielding SiO_2_ particles which are typically hundreds of nanometres to micrometres in diameter^7^,^8^. Luminescence usually only originates from such particles after high temperature annealing to remove quenching O-H groups. Natural hydrolysis of single source alkoxide precursors in controlled hydrolysis reactions can also produce RE-doped metal oxide nanomaterials^9^. Non-solution based routes involve ion implantation of lanthanide ions using sputtering techniques which allow strict control over the lanthanide concentration, though this technique is generally used to fabricate bulk glasses and thin films^10^.

Thermolysis is a well-known solid state chemistry technique for the preparation of (bulk) silica, alumina or titania based materials, which is often carried out by thermal decomposition of a complex or material (*e.g.* siloxanes or siloxides) in the presence of a silica or alumina support^11^,^12^,^13^,^14^,^15^,^16^. Thermolysis of inorganic molecules and clusters has also been shown to yield nanostructured materials^17^,^18^. Materials produced in this way can be doped with a variety of species, including metals, through the selection of appropriate precursors. The success of this method in producing well-defined bulk species and nanostructures is attributed to the rare occurrence of interparticle collisions in the solventless reaction environment, allowing particle growth to proceed primarily by monomer addition to the particle surface leading to monodisperse size and shape distributions^18^.

Siloxanes and silsesquioxanes can be used as ligands in the formation of a wide variety of interesting metallorganic complexes, for example hybrid complexes of lanthanides and oligosilsesquioxanes^19^,^20^,^21^. Metallasilsesquioxanes contain the functional group Si-O-M, where M can be a main group metal, *d*-transition metal or *f*-group element^21^,^22^,^23^,^24^,^25^. Their preparation and interesting properties have been described in several comprehensive reviews in recent years^26^,^27^,^28^,^29^,^30^ and they have been utilized as catalysts industrially^2^,^27^,^29^,^30^,^31^. RE metallasilsesquioxanes have great potential as precursors for new luminescent materials because siloxane ligands surrounding the RE metal centre can prevent the formation of RE-O-RE clusters that normally result in quenching by non-radiative recombination through energy dissipation and cross relaxation^32^. In addition, silsesquioxanes have structural similarities with silica (particle and bulk) materials, providing potential for synthesis of new silica based nanostructures^33^, and enabling the tailoring of surface structure^34^. Indeed, polyhedral oligomeric silsesquioxanes (POSS) have been used as a starting material to produce well-defined mesoporous materials^35^; luminescent Eu^3+^-containing mesoporous silicate nanostructures have been prepared *via* ionic incorporation of RE ions during the condensation reactions of POSS and have shown promise in bioimaging applications^36^. Pioneering work by Maxim *et al*. and Kenji *et al*. described the first examples of the formation of microporous amorphous silicate materials with well-distributed metal species *via* high temperature calcination of metallasiloxane species^37^,^38^,^39^. Herein, we report a convenient thermolytic route to the preparation of luminescent rare earth doped silica nanoparticles with uniform size by thermolysis of a new single source metallasilsesquioxane precursor.

An incompletely condensed trisilanol (*c*-C_6_H_11_)_7_Si_7_(OH)_3_ was initially prepared through the kinetically controlled hydrolysis of trichlorocyclohexylsilane (Fig. 1a) following well-known literature protocols^31^. ^29^Si NMR of (*c*-C_6_H_11_)_7_Si_7_(OH)_3_ showed peaks at −60.36, −68.25 and −69.79 ppm in a ratio of 3:1:3, representing the 3 environments of Si in the incompletely condensed trisilanol (Figure S1, Supplementary Information, SI)^31^. FTIR of (*c*-C_6_H_11_)_7_Si_7_(OH)_3_ (Figure S2, SI) additionally showed stretches representative of terminating hydroxyl groups (2800–3400 cm^−1^), Si-O-Si stretching and bending vibrations (1050–1195 cm^−1^) and Si-C stretches (between 1260 and 1450 cm^−1^)^40^,^41^. A europium precursor [(THF)_3_Li(*μ*-Cl)Eu[N(SiMe_3_)_2_]_3_] (Fig. 1b) was additionally prepared using a well-documented method^42^,^43^. ^29^Si NMR of this compound (Figure S3, SI) showed a single peak at 54.78 ppm, as dictated by the symmetry of the compound; FTIR spectroscopy (see Methods) showed peaks which were closely in agreement with literature^42^.

A europium-based metallasilsesquioxane compound (compound **1**) was prepared from these precursors following a silylamide route, reacting the incompletely condensed trisilanol (*c*-C_6_H_11_)_7_Si_7_(OH)_3_ with the europium precursor [(THF)_3_Li(*μ*-Cl)Eu[N(SiMe_3_)_2_]_3_] (Fig. 1c). The ^29^Si NMR of compound **1** displays a series of peaks (Fig. 2). A peak at 11.39 ppm is due to the presence of a small amount of vacuum grease in the sample. The peak at −21.67 ppm corresponds to a by-product of the reaction, likely [HN(SiMe_3_)_2_]. The insets of Fig. 2 display the peaks which are attributed to compound **1**. These peaks, at −69.50, −69.20, −67.90, −67.21, −67.15, −67.00, −66.70, −60.00, −58.37, −57.34, −55.61 ppm, are in an approximate ratio of 1:2:1:1:1:1:1:1:2:1:1. The two peaks which are near to one another and appear similar to a doublet signal (at −67.21 and −67.15 ppm) are close in proximity possibly due to a very fast transfer of a proton between two of the OH^−^ groups in the structure. The presence of THF molecules coordinated to the Eu-metal in the complex can cause a distortion of the shape and symmetry of the molecule. The 11 peaks present in the ^29^Si NMR are all associated with the suggested structure in Fig. 1c due to this lack of symmetry and are in agreement with similar structures which are reported in the literature^2^.

FTIR of compound **1** (Figure S4, SI) showed peaks as expected for the siloxane and hydrocarbon groups shown in the proposed structure and correspond well with stretches observed in similar silsesquioxane complexes reported in the literature^2^,^35^,^36^,^41^. Peaks at 2917 and 2848 cm^−1^ are due to CH stretches of the cyclohexyl substituent^41^. Stretches in the fingerprint region (600–900 cm^−1^) are due to C-C bonds of cyclohexyl groups. Peaks between 1015–1190 cm^−1^ are representative of Si-O-Si stretching and bending vibrations^40^,^41^. Si-C stretches can be observed between 1260 and 1450 cm^−1^ representative of asymmetric silicon-phenyl stretches^2^,^35^. The clear absence of OH^−^ groups when compared with the spectrum for the trisilanol (*c*-C_6_H_11_)_7_Si_7_(OH)_3_ (Figure S2, SI) corresponds with the proposed structure in Fig. 1c, due to the coordination of these groups to the RE metal (bridging mode).

Thermolysis of this rare earth metallasilsesquioxane was performed as a new route to produce luminescent rare earth doped silica nanoparticles. Thermogravimetric analysis (TGA) was initially carried out in order to assess the thermal stability of the prepared compound (Fig. 3). Mass loss between 150–235 °C is attributed to the removal of hydroxyl groups and coordinated solvent molecules in the structure. Siloxanes are reported to have a ‘melting’ phase between 270–300 °C^13^,^14^–this behavior is observed as small mass losses in the TGA profile in this region. The largest mass loss occurred between 350–600 °C, due to loss of hydrocarbon groups from the cyclohexyl substituents on the compound. Heating to 900 °C resulted in the formation of a black tar-like material, with <30% of total mass remaining, indicating significant compound degradation at this temperature. Previously, Tilley and co-workers have demonstrated that decomposition of single-source precursors (siloxy compounds) can occur at ~150 °C and can efficiently produce homogeneous metal-silica (bulk) materials^12^,^15^. With this in mind, and considering the thermal profile of compound **1** described above, thermolysis experiments were carried out at 300 °C under a nitrogen atmosphere (see Methods) and the resulting white powder was collected for analysis. Transmission electron microscopy (TEM) of **1** after thermolytic treatment demonstrated the presence of spherical nanoparticles with diameters of 13.5 ± 4.0 nm (Fig. 4). Energy dispersive X-ray spectroscopy (EDS) of the nanoparticles indicated their relative elemental composition (1:8:23 Eu:Si:O atom ratio, Table S1 and Figure S5, SI). This is at a slightly higher composition ratio than the precursor species (compound **1**) due to the rearrangement of silicon atoms during thermal treatment and the incorporation of multiple Eu centres in the particles.

FTIR spectroscopy of the thermolytically-produced nanoparticles (Figure S4, SI) shows peaks representing silicate groups, as expected, for example strong stretches between 1015–1190 cm^−1^ representing Si-O-Si stretching and bending vibrations^40^,^41^, and asymmetric silicon-phenyl stretches between 1260 and 1450 cm^−1^, though these appear reduced compared to the compound prior to thermolytic treatment^2^,^35^. Raman spectra of compound **1** before and after thermal treatment are shown in Fig. 5. Before thermolysis, the compound showed some sharp peaks indicative of symmetric and asymmetric vibrations of Si-O-Si bonds within the compound^44^. After thermolysis, several of these sharp peaks disappeared and broadened peaks emerge, indicative of amorphous silica, as expected, due to the nanoparticulate structure of the material post-thermolysis. Observed stretches are similar to similar metallic siloxane materials in the literature (produced *via* calcination), where peaks around 800 cm^−1^ are indicative of Si-O-Si stretches and those at ~600 and 485 cm^−1^ represent tri and tetracyclosiloxane rings of siloxanes^38^; the broadened peak centred at 360 cm^−1^ can be assigned to O-bending vibrations in the SiO_4_ tetrahedral silica matrix^45^. X-ray diffraction patterns collected of the thermolytically-produced nanoparticles (Figure S6, SI) demonstrated some crystalline-like peaks indicative of the presence of siloxane and hydrocarbon peaks, as expected due to the structure of the starting material. None of the peaks fit well with known structures in the JCPDS database, however, and the broad nature of several of these peaks is indicative of mostly amorphous silica based nanostructures (as observed in Fig. 4).

Solid state photoluminescence spectra taken before thermolysis demonstrate characteristic emission peaks as expected from Eu^3+^ ions present in the compound (Fig. 6a); with peaks at 578, 592, 612, 653 and 700 nm corresponding to the Eu^3+^ transitions: ^5^D_0_ → ^7^F_j_ (j = 0, 1, 2, 3, 4). This correlates well with other lanthanide-POSS species described in the literature, where characteristic Eu^3+^ emission is preserved in complex siloxane species and materials^32^,^36^. Herein, however, broad underlying emission is also present and appears significantly enhanced after thermolytic treatment and production of nanoparticles (Fig. 6b,c).

Broad emission in the visible range of the spectrum is well documented in bulk silica gels and nanostructures prepared using thermal and high energy treatments^45^,^46^,^47^,^48^. This emission, often present in species prepared without the addition of traditional organic or metallorganic dyes, is thought to originate from hydrocarbon-based defects^45^,^46^ derived from the hydrocarbon groups present on side-chains of the silicate precursors–in this case, the silsesquioxane compound. This emission is apparent prior to thermal treatment due to the high amount of hydrocarbon groups present in this asymmetric complex. The emission appears more strongly after thermal treatment, due to these species’ displacement and rearrangement in the nanostructures during strong heating. Whilst this broad emission masks some of the characteristic Eu^3+^ peaks, peaks at 612 and 700 nm still remain visible after thermal treatment, indicative of the remaining presence of the Eu^3+^ species. Broadening of Eu^3+^ emission peaks can also result from clustering and phonon-assisted energy transfer effects between adjacent Eu^3+^ ions in the Eu^3+^ clusters, present herein due to rearrangements of the silsesquioxane structure during thermal treatment^1^.

Thus, we have demonstrated that new rare earth based metallasilsesquioxane compounds can be used as single source precursors in the thermolytic preparation of rare earth-doped silica nanoparticles. The thermolytic approach presented herein is a convenient and promising technique for preparing silica nanoparticles from a single source precursor, yielding Eu^3+^-doped silica nanoparticles with small sizes and spherical structures. The siloxane structure of the parent species provides an appropriate inorganic cluster to allow the formation of small sized silicate nanoparticles. The nanoparticles retain the characteristic Eu^3+^ lanthanide luminescent properties of their parent compounds and a broad luminescent baseline due to the presence of hydrocarbon defects, which becomes enhanced by thermal treatment^45^,^46^,^47^. We believe that this approach could be used to prepare various nanostructured materials doped with rare earth and other metals (through changing the metal centre of the precursors used to prepare them) for a variety of potential optical, biomedical and other applications.

## Methods

### Materials and Methods

All starting materials have been supplied by Sigma-Aldrich unless stated otherwise. Anhydrous tetrahydrofuran (THF) was purified by heating under reflux for 2–3 hours over a sodium-potassium alloy and benzophenone, followed by distillation under argon and then condensation into a reaction flask. Benzophenone forms a blue ketyl once the solvent is dry.

NMR spectra were collected from Bruker 400-Avance-3 (9.4 T) spectrometer with samples prepared in C_6_D_6_ under a N_2_ atmosphere and analyzed immediately after preparation. A JEOL JEM-2100, 200 kV LaB_6_ transmission electron microscope operated at 120 kV with a beam current of ~65 mA was used to image nanoparticle samples. Aqueous suspensions were drop-cast onto a formvar coated copper grid for imaging. Size analysis was carried out using ImageJ software by measuring a minimum of 100 particles. An energy dispersive X-ray spectroscopic (EDS) system attached to a JEOL JEM-2100 transmission electron microscope was used to collect elemental data on the prepared nanoparticles; EDS was carried out on several areas of the TEM sample, sampling a minimum of 100 particles. FTIR spectra were recorded by diffuse reflectance in dry Nujol (dried over molecular sieves) between NaCl discs using a Perkin Elmer Spectrum One FT-IR spectrophotometer. Raman spectra and solid state photoluminescence spectra were measured with a Renishaw 1000 micro-Raman system with a Leica microscope. The excitation wavelength was 457 nm from an Ar^+^ ion laser (Laser Physics Reliant 150 Select Multi-Line) with a typical laser power of ~10 W cm^2^. Samples were placed as a solid powder on a silicon substrate and pressed flat using manual pressure. The laser was focussed on the sample, with a spot size of ~10 μm and emission was collected. As such, the data presented should not be taken as quantitative and directly comparable between samples in terms of emission intensity. Thermogravimetric analysis (TGA) and thermolysis was carried out using a Perkin Elmer Pyris 1 TGA machine operated with a heating rate of 10 °C/min under an atmosphere of N_2_. X-ray diffraction was performed using an Empyrean diffractometer equipped with a Co Kα lamp fitted with a beam knife, 6 h scans were carried out measuring 5–50 degrees 2θ. Bright field and fluorescence microscope images were taken using an Olympus CKX41 optical system fitted with an Olympus XC30 camera and CoolLED pE300-white light source. Images were taken with a x10 objective lens under bright field (6 V, 30 W halogen lamp) or excited with 365 nm wavelength (operated at 80 W). Camera settings were set at 371.6 ms exposure time with 4.9 dB gain and 2080 × 1544 resolution.

### Preparation of (*c*-C_6_H_11_)_7_Si_7_(OH)_3_

The preparation of (*c*-C_6_H_11_)_7_Si_7_(OH)_3_ was carried out according to published procedure^31^.

^**29**^**Si (79.5 MHz, C**_**6**_**D**_**6**_**, 25 °C):** δ = −69.79, −68.23, −60.36 ppm.

^**1**^**H (400 MHz, C**_**6**_**D**_**6**_**, 25 °C):** δ = 7.28, 2.27, 2.25, 1.93, 1.77, 1.76, 1.74, 1.72, 1.47, 1.45, 1.43, 1.41, 1.39, 1.37, 1.19, 1.17, 1.15, 0.99, 0.58, 0.41 ppm.

^**13**^**C NMR (100 MHz, C**_**6**_**D**_**6**_**, 25 °C):** δ = 27.54, 27.51, 27.38, 27.13, 27.05, 27.02, 26.93, 26.89, 26.84, 24.24, 23.91, 23.42 ppm.

**IR (Nujol, cm**^**−1**^): 3149 (br), 1445 (s), 1286 (s), 1193 (s), 1080 (s), 890 (s), 846 (w), 824 (w), 750 (w), 677 (w).

### Preparation of [(THF)_3_Li(*μ*-Cl)Eu[N(SiMe_3_)_2_]_3_]

**Figure 1:**
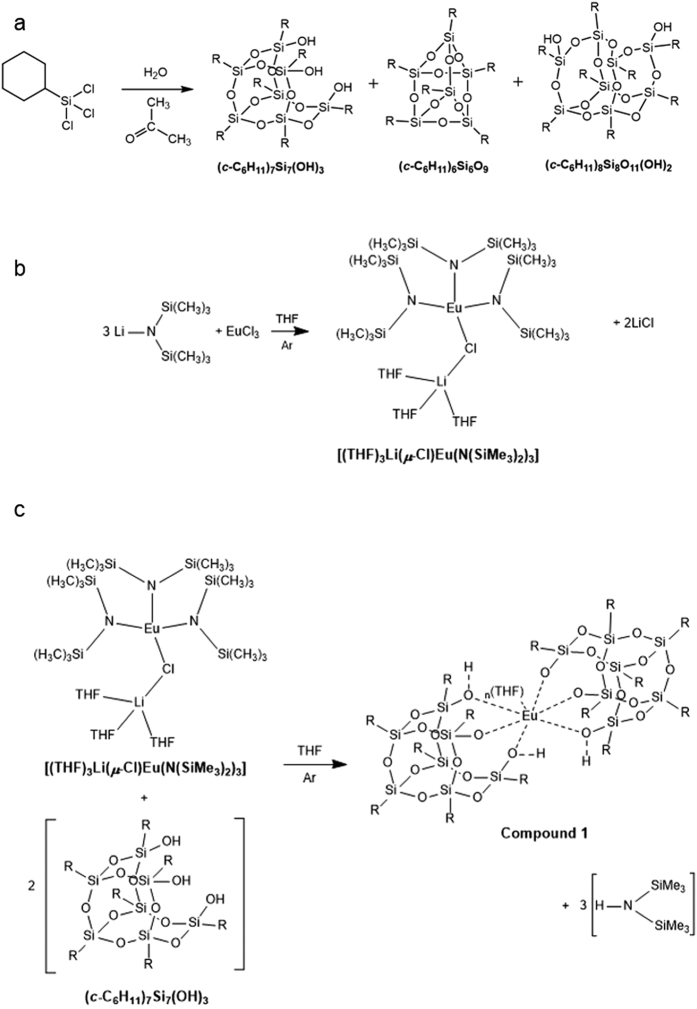
(**a**) Schematic representation of the preparation of incompletely condensed and completely condensed silsesquioxanes prepared through the kinetically controlled hydrolysis of trichlorocyclohexylsilane; (**b**) Preparation of [(THF)_3_Li(*μ*-Cl)Eu[N(SiMe_3_)_2_]_3_] ligand; (**c**) Preparation of compound **1**, where R = cyclohexyl groups.

Anhydrous europium chloride (0.34 g, 1.31 mmol) was dissolved in anhydrous THF (30 mL). Li(N(SiMe_3_)_2_) (1.04 g, 4.3 mmol) was gradually added to the stirring solution at room temperature. The reaction was stirred at room temperature for several days. The white precipitate was allowed to settle before filtration of the orange liquid containing [(THF)_3_Li(*μ*-Cl)Eu[N(SiMe_3_)_2_]_3_] (Fig. 1b). The orange liquid was dried under vacuum to a red/orange powder which was then characterized.

^**29**^**Si (79.5 MHz, C**_**7**_**H**_**8**_**, 25 °C):** δ = 54.78 ppm.

**IR (Nujol, cm**^**−1**^): 2880 (w), 1405 (w), 1240 (s), 1180 (s), 1040 (m), 979 (m), 827 (m) 766 (m), 750 (m), 671 (w).

### Preparation of compound 1

(*c*-C_6_H_11_)_7_Si_7_(OH)_3_ (Fig. 1c, 0.14 g, 0.13 mmol) was dissolved in anhydrous THF (50 mL). [(THF)_3_Li(*μ*-Cl)Eu[N(SiMe_3_)_2_]_3_] (0.04 g, 0.063 mmol) was slowly added to the solution with stirring at room temperature. The solution was refluxed under argon at 70–80 °C for two days. The pale yellow liquid containing compound **1** (Fig. 1c) was dried under vacuum to yield a pale yellow powder which was characterized.

^**1**^**H (400 MHz, C**_**6**_**D**_**6**_**, 25 °C):** δ = 7.27, 2.20, 1.86, 1.69, 1.37, 1.33, 0.97, 0.38 ppm.

^**13**^**C NMR (100 MHz, C**_**6**_**D**_**6**_**, 25 °C):** δ = 27.52, 27.11, 26.87, 24.96, 24.21, 23.76, 23.37, 1.49, 0.96 ppm.

^**29**^**Si (79.5 MHz, C**_**4**_**H**_**8**_**O, 25 °C):** δ = −69.50, −69.20, −67.90, −67.21, −67.15, −67.00, −66.70, −60.00, −58.37, −57.34, −55.61, −21.67, 11.39 ppm.

**IR (Nujol, cm**^**−1**^): 2917 (w), 2848 (w), 1445 (w), 1260 (s), 1190 (s), 1080 (s), 1015 (s), 890 (s). 796 (s).

### Preparation of nanoparticles

Compound **1** was placed in a ceramic boat and heated under an atmosphere of N_2_ at a heating rate of 10 °C/min using a Perkin Elmer Pyris 1 thermogravimetric analysis machine and held at 300 °C for 30 mins. Samples were retained after burning for characterization as described in the main text.

### Data Availability

Datasets are available open access at http://wrap.warwick.ac.uk/85408/.

## Additional Information

**How to cite this article:** Davies, G.-L. *et al*. Rare Earth Doped Silica Nanoparticles *via* Thermolysis of a Single Source Metallasilsesquioxane Precursor. *Sci. Rep.* **7**, 45862; doi: 10.1038/srep45862 (2017).

**Publisher's note:** Springer Nature remains neutral with regard to jurisdictional claims in published maps and institutional affiliations.

## Supplementary Material

Supplementary Information

## Figures and Tables

**Figure 2:**
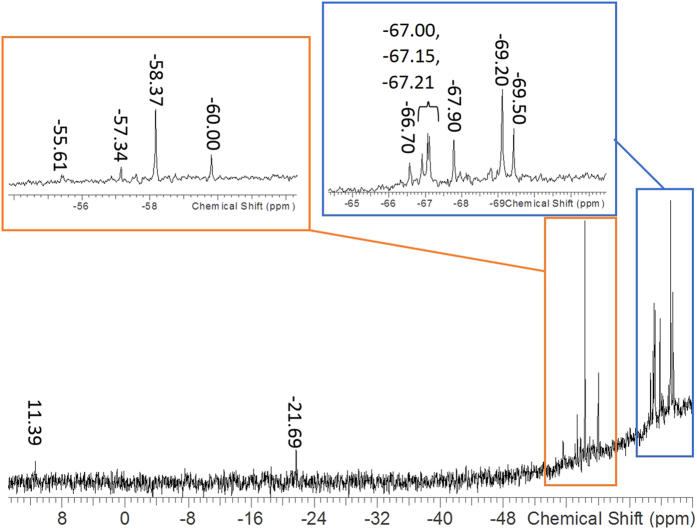
^29^Si NMR of compound **1**, showing peaks at −69.50, −69.20, −67.90, −67.21, −67.15, −67.00, −66.70, −60.00, −58.37, −57.34, −55.61, −21.67, 11.39 ppm, insets show close-up of the peaks representing its structure.

**Figure 3:**
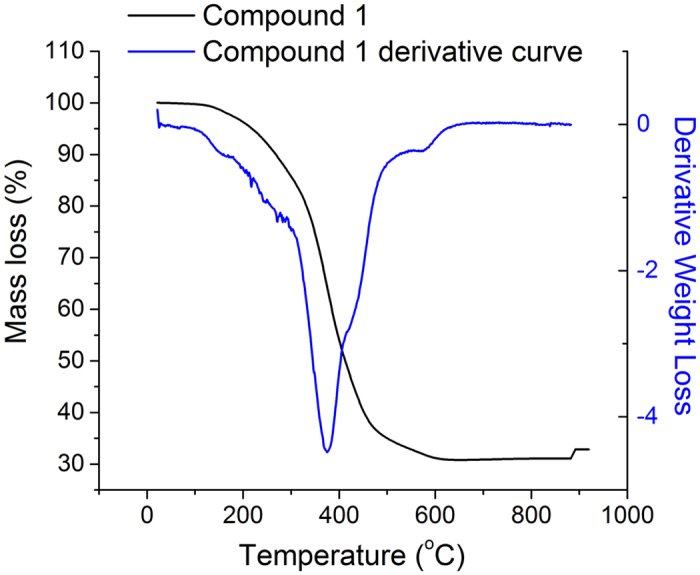
Thermogravimetric analysis curve (black) and derivative curve (blue) of compound **1** heated under an atmosphere of N_2_.

**Figure 4:**
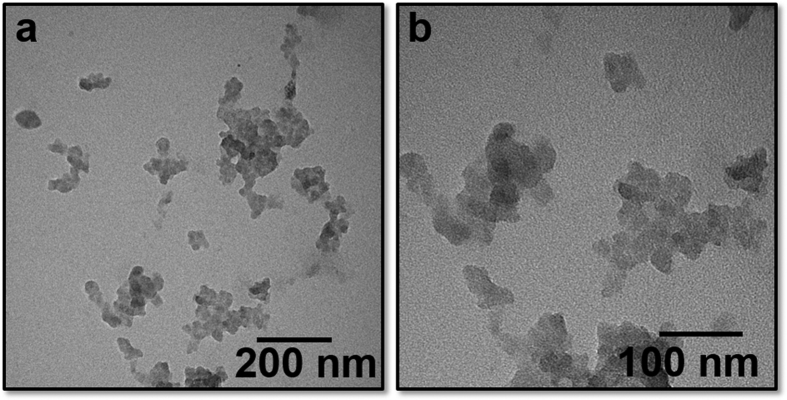
Transmission electron microscope images of nanoparticles (13.5 ± 4.0 nm) formed by the thermolysis of compound **1** at 300 °C under a N_2_ atmosphere.

**Figure 5:**
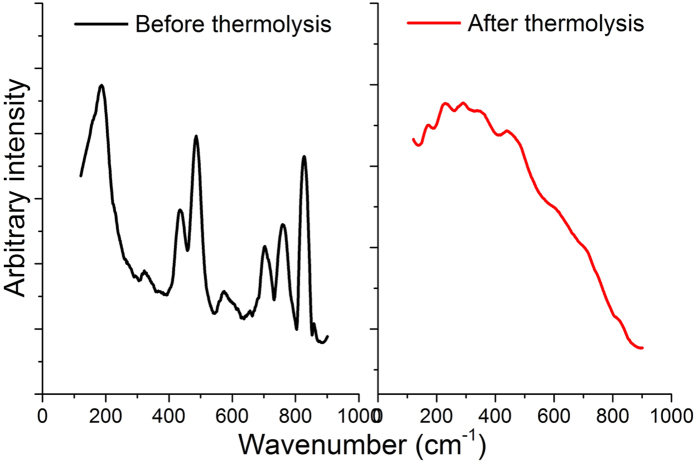
Raman spectra of compound **1** before (black) and after (red) thermolysis at 300 °C under N_2_ atmosphere.

**Figure 6:**
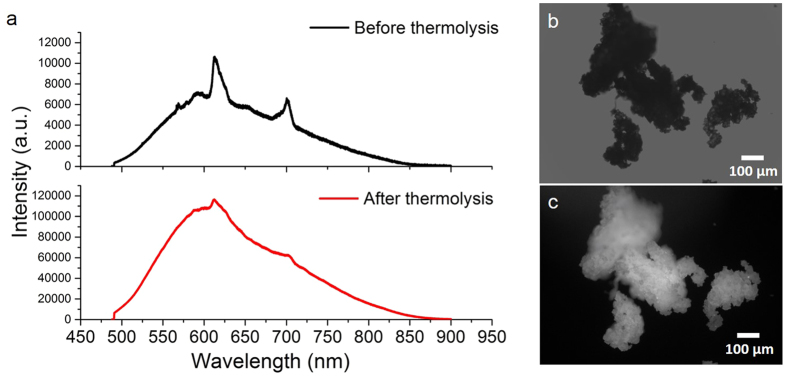
(**a**) Solid state photoluminescence spectra of compound **1** before (black) and after (red) thermolytic treatment, (**b**) bright field and (**c**) fluorescence (*λ*_*ex*_ 365 nm) microscope images of compound **1** following thermolysis.
