# Supplementary material for: Rare Earth Doped Silica Nanoparticles via Thermolysis of a Single Source Metallasilsesquioxane Precursor
Source: Sci Rep. 2017 Apr 5;7:45862. doi: 10.1038/srep45862 (PMC5381092; doi:10.1038/srep45862)
Supplement: Supplementary Information [file srep45862-s1.pdf]

Rare Earth Doped Silica Nanoparticles *via* Thermolysis of a Single Source Metallasilsesquioxane Precursor

Gemma-Louise Davies[a*], John O'Brien[b], and Yurii K. Gun'ko[*b,c]

[a] Department of Chemistry, University of Warwick, Coventry CV4 7AL, UK.

[b] School of Chemistry and CRANN Institute, Trinity College Dublin, Dublin 2, Ireland.

[c] St. Petersburg National Research University of Information Technologies, Mechanics and Optics, 197101, St. Petersburg, Russia.

* g-l.davies@warwick.ac.uk and igounko@tcd.ie

Supplementary Information

Figures and Tables

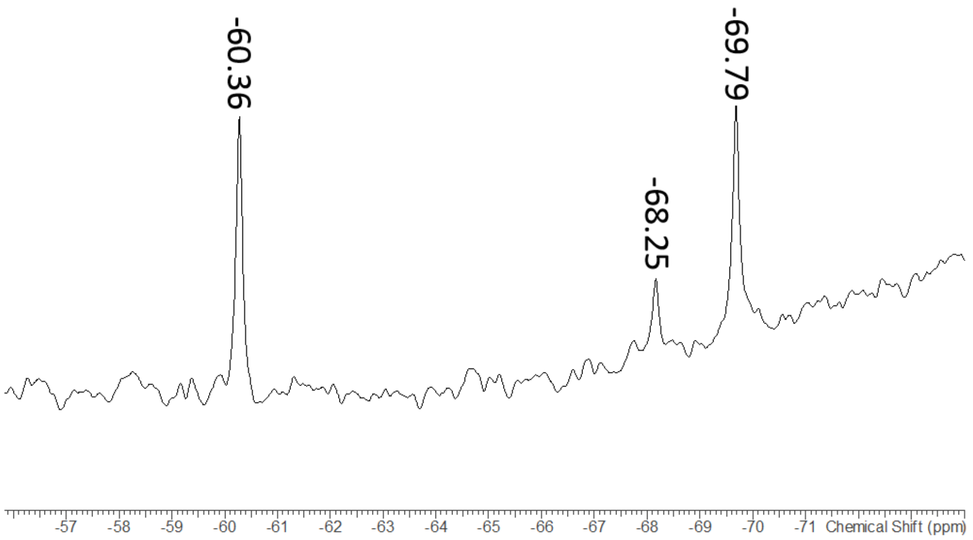

Figure S1. ^{29}Si NMR of $(c\text{-}C_6H_{11})_7Si_7(OH)_3$ showing peaks at -60.36, -68.25 and -69.79 ppm in a ratio of 3:1:3, representing the 3 environments of Si in the incompletely condensed trisilanol.

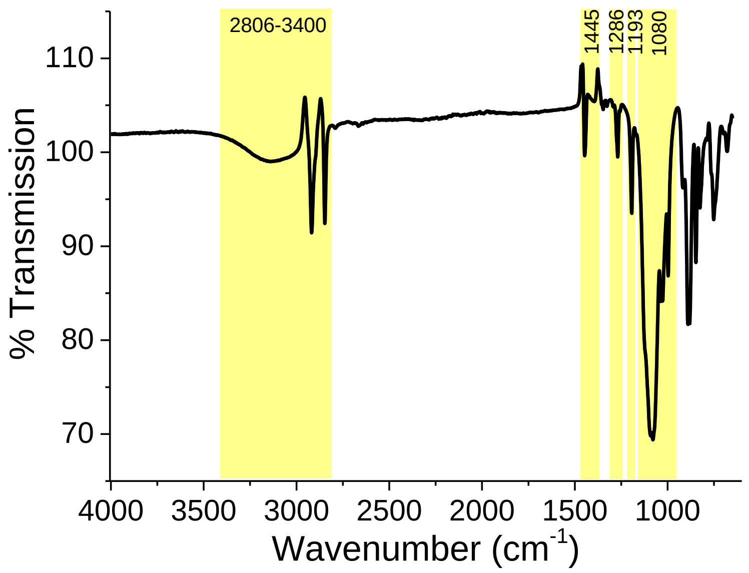

Figure S2. FTIR of (c-$C_6H_{11})_7Si_7(OH)_3$.

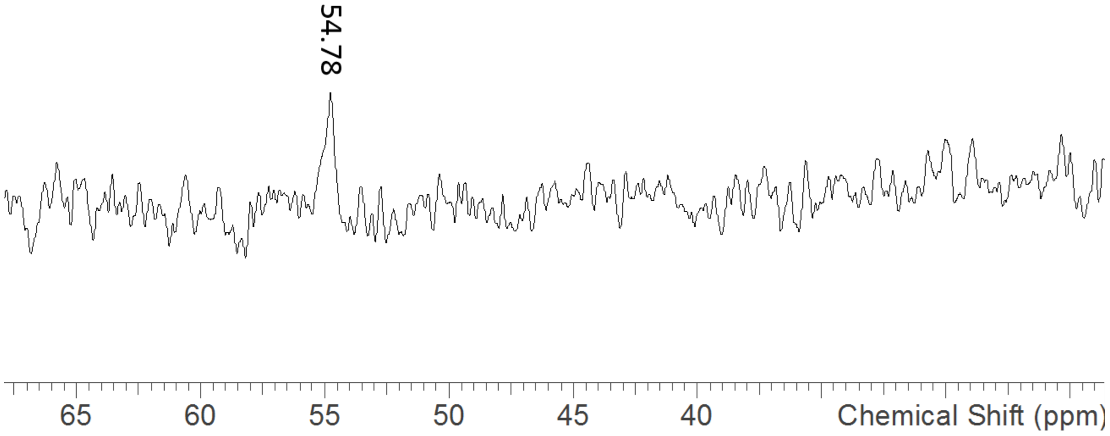

Figure S3. ^{29}Si NMR of [(THF)$_3$Li(μ-Cl)Eu[N(SiMe$_3$)$_2$]$_3$] showing a single peak at 54.78 ppm, as dictated by the symmetry of the compound.

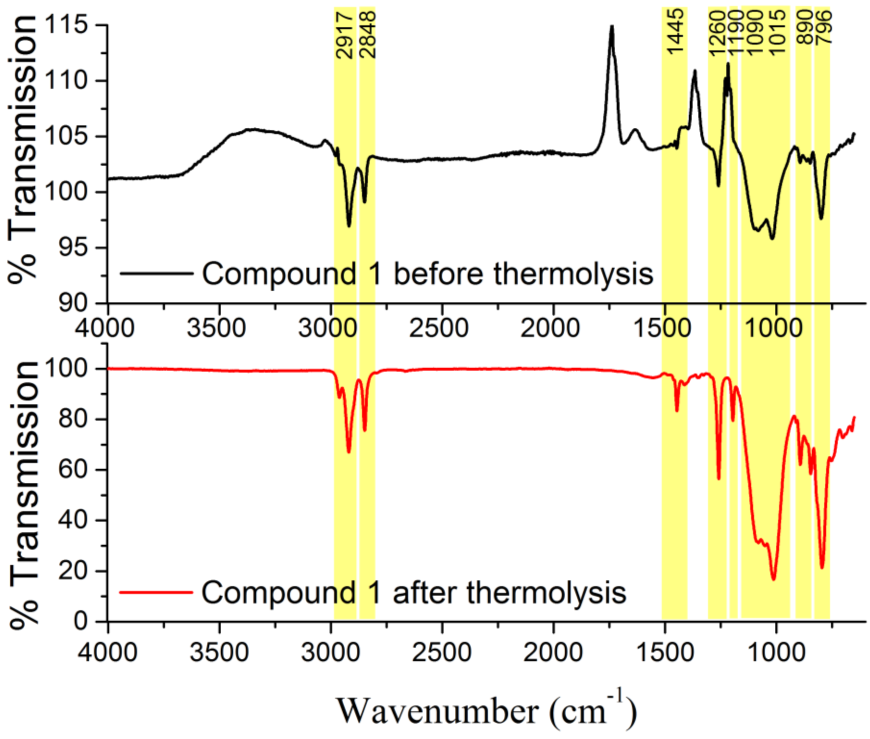

Figure S4. FTIR of compound **1** before and after thermolysis, as labelled.

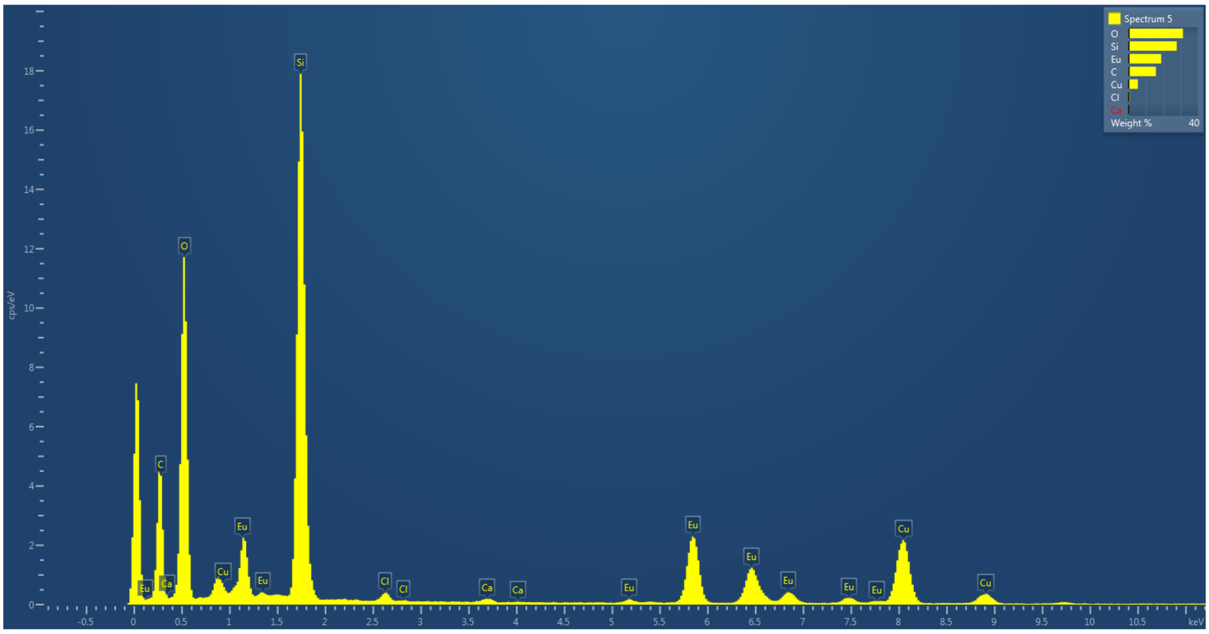

Figure S5. EDS spectrum showing elements present in thermolytically-prepared nanoparticles.

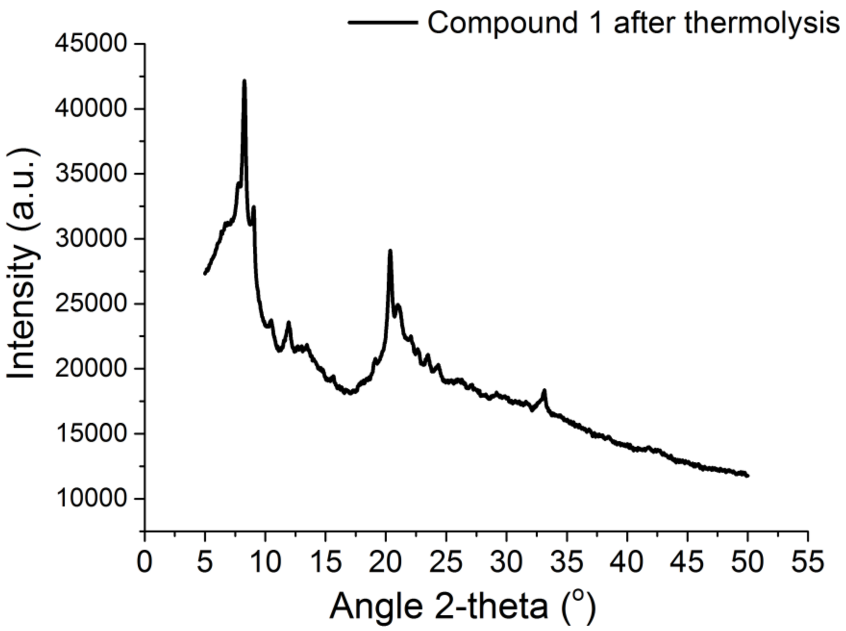

Figure S6. X-ray diffraction pattern of nanoparticles produced after thermolytic treatment of compound **1**.

Table S1. Energy dispersive x-ray spectroscopy(EDS) data showing elemental composition of nanoparticles prepared by the thermolytic decomposition of compound **1**.

Element	%Atom
C [a]	29.21
O	43.65
Si	22.01
Cu [b]	1.86
Eu	2.75

[a] C signal comes predominantly from the carbon support film; [b] The presence of Cu is due to the copper grid used to support sample.
